# Annotations

**Published:** 1902-05-17

**Authors:** 


### ANNOTATIONS.

### The Midwives Bill.

THE Midwives Bill occupies so good a position in the list of private Bills, that if all goes well with it there is a fair chance of its being read a third time on June 13. Whether so far as its public utility is concerned it is worth all the fuss that has been bestowed upon it is a question which will be answered very differently by different people. Those who consider that by improving the legal status of the certified midwife they will improve the conditions under which children are brought into the world in the houses of the poor, will no doubt be full of hope that the Bill will be all powerful for good. Those, on the other hand, who feel, as many of those who have most carefully considered the matter do feel, that all this establishment of a privileged class of midwives, without setting up any machinery to prevent the continuance of the old evils which have been so continually paraded as the reason and excuse for demanding legislation, renders the whole scheme somewhat of a sham. What is really wanted for the protection of poor women in childbirth is not the establishment on a legal basis of an interesting and sometimes lucrative profession for women, desirable as this may be, but a firm enactment putting a stop to the practice of midwifery by any except those who possess that minimum of knowledge and training which the Board shall consider essential; and this is just what the Bill fails to provide. That the Bill will do harm to the medical profession as some profess to fear, we do not believe; in fact, it is not impossible that its provisions may be found a considerable assistance by many elderly practitioners who, when midwives are legally qualified, will not hesitate to employ them as assistants in that particular branch of their practice. It is not because the Bill is likely to do any particular harm that we think so poorly of it, but because those who framed it have missed the very point on which they went before the public, and for the sake of getting a bill of any kind have been willing to accept one which leaves the poor woman nearly as much as ever at the mercy of the ignorant, untrained, and dirty midwife of whom at the beginning of the discussion we heard so much.

### An Army Medical College for London.

AMONG the various proposals which are in the air in connection with the reform of the Army Medical Service is the scheme of the Army Board for the establishment of a special military medical school in London. There is much to be said both for and against such a plan. To many London teachers it appears that there are already hospitals enough and schools enough, and that there is teaching power even in excess of all the possible requirements of the Army Medical Service. Moreover, it is sufficiently obvious that if the period of special study which is to be allowed in all cases before entrance for the examination for promotion to the rank of major is to become of real service it must be to a large degree, if not entirely, spent in the great civil hospitals of London. There seems, then, at first sight but little excuse for the establishment of a special college. It has, however, to be remembered that the field of knowledge required by the officers of the R.A.M.C. extends over a very different area from that which is taken cognisance of by the qualifying examining bodies. This is especially the case in regard to bacteriology and hygiene, in both of which, again, the knowledge required is and must be a good deal specialised to meet Army requirements, and although we have no donbt that many of the existing schools would be ready to take these officers through any special syllabus which might be laid down it can hardly be questioned that for the sake of economy of time, if not also of money, a Government college for the training of Army, Navy, and perhaps of colonial medical officers, situated in London and staffed not exclusively by Army professors, might prove the most effectual means of enabling officers to make the most of their study leave—that is to say, so far as college work is concerned. Hospital practice is another affair; nothing can take away the necessity of their attending in the wards and theatres of the civil hospitals. There is one drawback in the scheme. It would almost certainly involve the destruction of the Netley School—which would be a great pity.

### Inebriate Women.

THE record of a home for inebriate women is always a sad story, and that of the home established by the Corporation of Glasgow is no exception to the rule. The home is Girgenti, an old mansion house, in a pleasant part of the country, so the surroundings are pleasant, and the discipline also is kind. But unfortunately, the Inebriates Act is such that the victims of the alcohol habit cannot as a rule be sent to a home without their own consent until practically all hope of their recovery is past. Men drink openly, but when a woman has come to the point of being four times within a year convicted in a police court for drunkenness, one may be sure that the habit is firmly established and that she has been drinking in secret for a long time. This is just what the superintendent at Girgenti found. As far as age goes, the inmates might not have been regarded as past hope of reformation, for of the 46 women received in the course of the year, 33 were under 40 years of age, one being a girl of 21. On the other hand, the authorities sent to the home a woman of 61, who might reasonably have been looked upon as past improvement. It cannot be said that the report of the year's working shows much ground for hope. Of the 46 who were received into the home, 39 were there at the end of the year. Of the remaining seven, one had died from accidental burning, one was in a hospital in Glasgow, one in the State Inebriate Reformatory at Perth, one in a lunatic asylum, two in prison, and one out on license as a domestic servant. Thus only one could be regarded as in any degree hopeful. The committee are of opinion that very few of the women who come under the scope of the Inebriates Act are of such a character and disposition as to give much ground for expecting that, even if they were cured of their vice, they would be able to support themselves. In the opinion of Dr. Cunningham, the medical superintendent, many of those admitted occupy, on the most lenient supposition, a borderland between sanity and insanity; indeed there is no doubt that inebriety is often closely associated with some forms of insanity.

